# Human and Deep Learning Predictions of Peripheral Lung Cancer Using a 1.3 mm Video Endoscopic Probe

**DOI:** 10.1111/resp.70057

**Published:** 2025-05-28

**Authors:** Edoardo Amante, Robin Ghyselinck, Luc Thiberville, Rocco Trisolini, Florian Guisier, Valentin Delchevalerie, Bruno Dumas, Benoît Frénay, Inès Duparc, Nicolas Mazellier, Cecile Farhi, Christophe Jubert, Mathieu Salaün, Samy Lachkar

**Affiliations:** ^1^ Department of Pneumology Rouen University Hospital Rouen France; ^2^ Department of Pulmonary Medicine and Interventional Pulmonology Catholic University of the Sacred Hearth, Fondazione Policlinico Universitario Agostino Gemelli IRCCS Rome Italy; ^3^ Faculty of Computer Science, NaDI University of Namur Namur Belgium; ^4^ Department of Pulmonology and Inserm CIC‐CRB 1404 Normandie Univ, UNIROUEN, LITIS Lab QuantIF Team EA4108, CHU Rouen Rouen France

**Keywords:** artificial intelligence, bronchoscopy, deep learning, imaging, peripheral pulmonary nodules, radial‐EBUS

## Abstract

**Background and Objective:**

Iriscope, a 1.3 mm video endoscopic probe introduced through an r‐EBUS catheter, allows for the direct visualisation of small peripheral pulmonary nodules (PPNs). This study assessed the ability of physicians with different levels of experience in bronchoscopy, and the ability of artificial intelligence (AI) to predict the malignant nature of small PPNs during Iriscope peripheral endoscopy.

**Methods:**

Patients undergoing bronchoscopy with r‐EBUS and Iriscope for peripheral PPNs < 20 mm with a definite diagnosis were analysed. Senior and Junior physicians independently interpreted video‐recorded Iriscope sequences, classifying them as tumoral (malignant) or non‐tumoral, blind to the final diagnosis. A deep learning (DL) model was also trained on Iriscope images and tested on a different set of patients for comparison with human interpretation. Diagnostic accuracy, sensitivity, specificity, and F1 score were calculated.

**Results:**

Sixty‐one patients with small PPNs (median size 15 mm, IQR: 11–20 mm) were included. The technique allowed for the direct visualisation of the lesions in all cases. The final diagnosis was cancer for 37 cases and a benign lesion in 24 cases.

Senior physicians outperformed junior physicians in recognising tumoral Iriscope images, with a balanced accuracy of 85.4% versus 66.7%, respectively, when compared with the final diagnosis. The DL model outperformed junior physicians with a balanced accuracy of 71.5% but was not superior to senior physicians.

**Conclusion:**

Iriscope could be a valuable tool in PPNs management, especially for experienced operators. Applied to Iriscope images, DL could enhance overall performance of less experienced physicians in diagnosing malignancy.

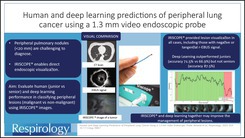

## Graphical abstract

Direct endoscopic visualisation of peripheral pulmonary nodule smaller than 2 cm is feasible using a miniaturised video endoscopy probe, and this endoscopic imaging of these peripheral lung lesions can predict malignancy with high accuracy, both by human eyes and a deep learning generated prediction model.

## 1 Introduction

Peripheral pulmonary nodules (PPNs) have become increasingly accessible for bronchoscopic sampling, due to advancements in peripheral bronchoscopy techniques [1] such as radial endobronchial ultrasound (r‐EBUS), electromagnetic or non‐electromagnetic navigation bronchoscopy, and robotic‐assisted bronchoscopy. However, diagnosing PPNs endoscopically remains challenging [2, 3], particularly for small PPNs, that is, those less than 20 mm in diameter [4, 5].

A critical step in peripheral bronchoscopy is optimising the sampling phase. Even after identifying and accessing the distal bronchus leading to the target during CT planning and navigation [6], it is essential to confirm the correct location of the sampling device [7].

While current verification tools such as r‐EBUS, fluoroscopy, cone‐beam CT, and digital tomosynthesis help reduce *body‐to‐scan divergence* [8, 9, 10], none of these techniques provide direct endoscopic visualisation of the peripheral lung lesion before sampling.

In a previous study [11], we used a miniaturised 1.3 mm video endoscopic probe (Iriscope, *Lys Medical, Charleroi, Belgium*) with r‐EBUS for direct visualisation of PPNs, including small subpleural lesions, to ensure precise localisation of the biopsy forceps. This approach achieved an 87% diagnostic rate and visually differentiated benign from cancerous peripheral nodules with a 93% positive predictive value. However, a limitation was that expert endoscopists performed the image interpretation, raising concerns about whether less experienced bronchoscopists could similarly recognise tumoral features [12, 13, 14, 15].

Artificial Intelligence (AI) has shown promise in improving lung nodule management [16], although challenges remain [17, 18]. Its role in the direct visualisation of small PPNs, however, is still unexplored.

This study aimed to assess the Iriscope's role in the endoscopic diagnosis of small PPNs, particularly in predicting malignancy. We evaluated whether its performance depends on physician experience and if integrating AI to the procedure could enhance diagnostic accuracy for both experienced and less experienced bronchoscopists.

## 2 Materials and Methods

### 2.1 Study Population

This single‐centre study was conducted at Rouen University Hospital between November 2023 and September 2024. All consecutive patients with PPLs < 20 mm who underwent bronchoscopy with r‐EBUS + Iriscope and had a definite diagnosis were analysed.

Clinical data, thoracic imaging, and pathology results were retrospectively reviewed. CT scans from the hospital's Picture Archiving Communication System included details on lesion type (ground glass, solid, sub‐solid), size, bronchus sign, and distance to the pleura.

The study protocol was approved by the Institutional review Board of Rouen University Hospital (E2024‐72). Research followed the European Directive 2014/536/EU and the French law 2012–300 on biomedical research. Consent was not required for retrospective data analysis under French law.

### 2.2 Bronchoscopy Procedure

The endoscopic route to the lesion was planned using virtual bronchoscopy navigation software (*LungPoint* planner, Broncus Medical Inc., San Jose, California, USA). Bronchoscopy was performed under local or general anaesthesia using a bronchoscope with a 4.2 mm outer diameter and a 2 mm working channel (BF‐P190, *Olympus, Tokyo, Japan*). Once the distal bronchus leading to the lesion was reached, the r‐EBUS probe (1.4 mm UM‐S20‐17S probe, Olympus *Tokyo, Japan*) was introduced via the guide sheath (1.9 mm guide sheath, K401, Olympus *Tokyo, Japan*) as described [6, 11]. After obtaining r‐EBUS images, the ultrasound probe was removed, and the Iriscope probe was advanced into the guide sheath to confirm lesion visualisation and sampling. The Iriscope procedure was systematically video‐recorded for further analysis. Cytological brushing and biopsy were performed through the guide sheath without repositioning to ensure sampling accuracy. Chest radiographs were not routinely performed post procedure. No rapid on‐site examination was available (Video 1).

**VIDEO 1:** Procedure diagnosis for a peripheral lung nodule with r‐EBUS + Iriscope. Video content can be viewed at https://onlinelibrary.wiley.com/doi/10.1111/resp.70057

Lung cancer diagnosis was based on the cytological or histological results of endoscopic sampling, CT‐guided biopsy, or surgery. Benign lesions were confirmed by negative biopsy with regression on CT follow‐up or microbiological findings responsive to treatment.

### 2.3 Human Interpretation

Iriscope videos were retrospectively analysed by two groups of endoscopists who did neither perform nor see the procedures, and were blind to the final diagnosis and the patient clinical history:

- Group 1: Two senior physicians, 2 physicians with > 10 years bronchoscopy experience;
- Group 2: Four junior physicians with < 2 years bronchoscopy experience.

**FIGURE 1:**
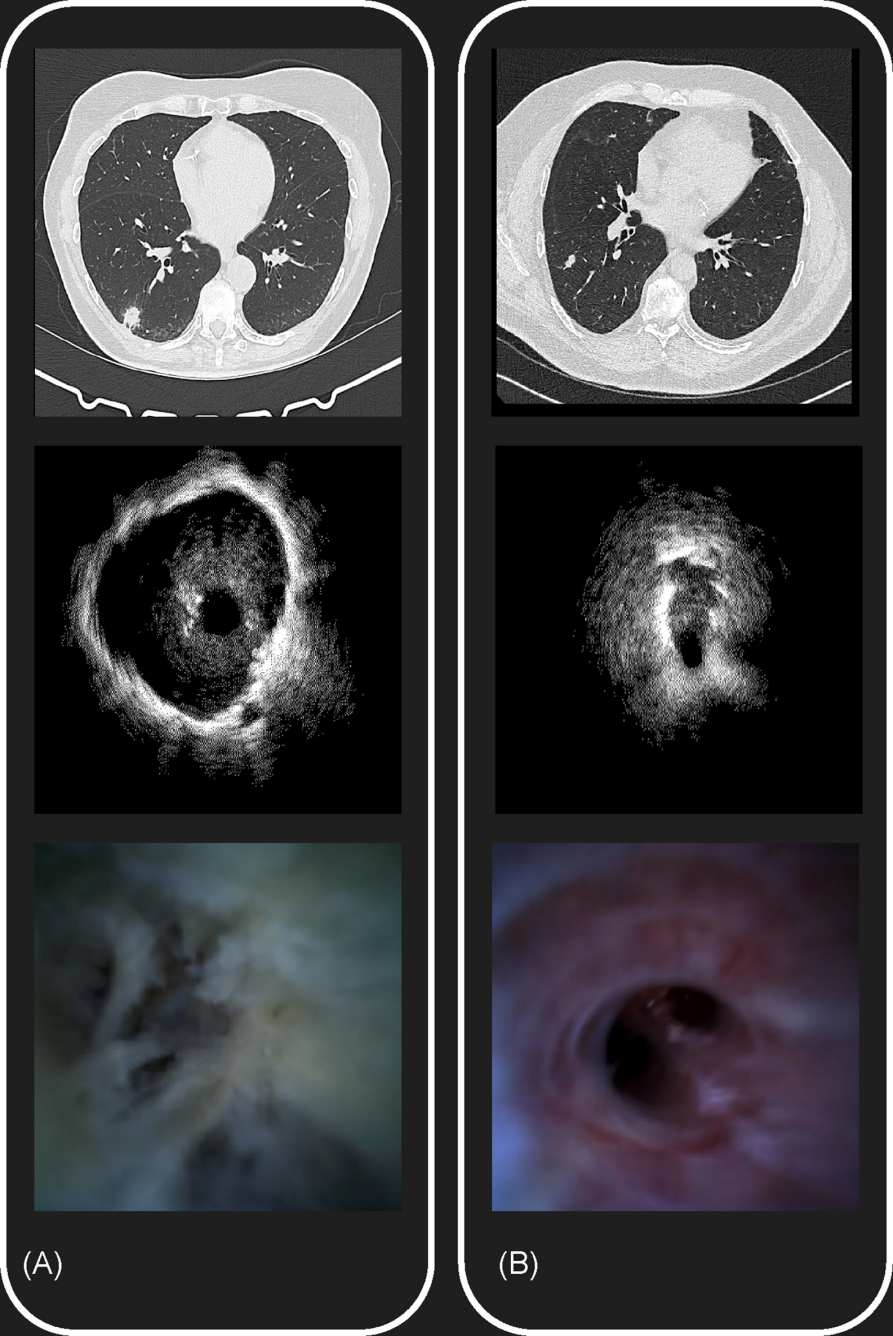
CT, r‐EBUS and Iriscope appearance of a 19 mm subpleural lung cancer and a 7 mm benign lesion. (A) Upper panel: CT scan image of a right lower lobe nodule; middle panel: Centred r‐EBUS image of the nodule; Lower panel: Tumoral aspect with ‘fish flesh’ appearance on Iriscope (final diagnosis: Adenocarcinoma). (B) Upper panel: CT scan image of a right lower lobe nodule; middle panel: Tangential r‐EBUS image of the nodule; lower panel: Normal parenchyma aspect on Iriscope (final diagnosis: Infection).

Based on endoscopic patterns established in our previous study [11], videos were classified as malignant (whitish friable tissue and/or mucosal outgrowth and/or stenosis) or benign (inflammation and/or secretions and/or normal bronchus appearance) (Figure 1).

### 2.4 Artificial Intelligence and Deep Learning

#### 2.4.1 Image Processing

Iriscope video recordings were anonymized, split into 62,072 frames (30 frames/s of 400 × 400 pixels), and grouped by patient (61 videos). The ResNet‐50 model [19], a convolutional neural network model, was pre‐trained on the ImageNet dataset [20], and was used for binary classification (malignant versus benign) of individual frames extracted from the videos.

Patients were randomly allocated into training (41 patients) and test (20 patients) sets, maintaining a 70/30 ratio (training/test sets) of malignant cases. Frames of the video sequences from each patient were labelled as malignant or benign by an expert endoscopist, who classified them based on the endoscopic patterns described above [11]. This human annotation, on top of the cancer/non‐cancer allocation of the patient, was only used for the purpose of training the DL model.

#### 2.4.2 Deep Learning Model Training and Initialization

Data augmentation (resizing, cropping, rotation, flips, application of shearing and Gaussian blur, etc.) were applied to improve model performance. This helped increase the variability of the dataset, thus improving the model's performance [21].

Since each of the datasets had an imbalance between total tumoral and non‐tumoral frames, a balanced sampling technique was used during training. In order to improve the robustness of the results, five independent DL models were trained on the whole training set, differing only in their initialization process [22].

#### 2.4.3 Testing and Evaluation of the DL Model

The DL model was evaluated using the test set, composed of all the patient's frames that were not included in the training process. This ensured that the model's performances were assessed on unseen data from unseen patients.

#### 2.4.4 Different Windows Size for DL Per‐Patient Interpretation

**FIGURE 2:**
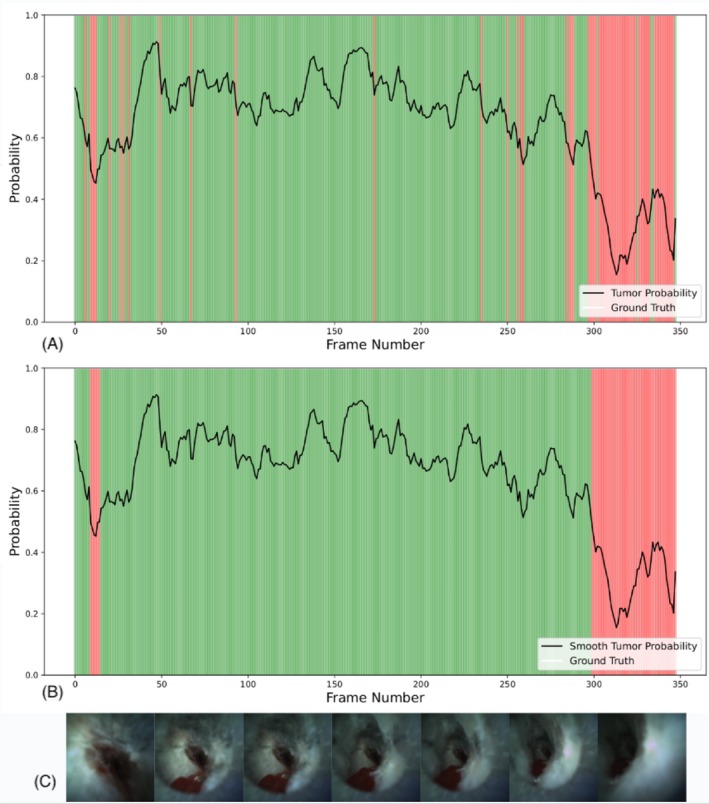
Frame by frame deep learning prediction on a cancer patient. (A) Illustration of model's predicted probability for cancer (y‐axis) against frame number position in the video sequence (x‐axis). Areas in green indicate a correct prediction by the model (probability superior to 50%), whereas areas in red indicate a wrong prediction. (B) The same information, with smoothing on the probability using ARIMA. (C) Representative Iriscope images of the patient during the procedure.

A sliding window approach (1 to 300 frames, with 1 frame corresponding to about 1/30th of a second) was used to generate per‐patient predictions, with frame sequences indicating “tumoral” if all frames in the window indicated a tumour. Window sizes of 30, 45, and 60 frames (1–2 s) were tested, reflecting typical video review by physicians. The ARIMA method [23] was used to smooth frame‐by‐frame predictions, improving stability (Figure 2).

Appendix A1 provides details on the artificial intelligence and deep learning methods applied in the present study.

## 3 Statistical Analysis

Analysis was conducted using IBM *SPSS Statistics* version 29.0.2.0 and *Python 3.10* with the following libraries: *scipy 1.14.1; scikit‐learn 1.5.1; Pandas 2.2.2* and *Numpy 2.1.0*.

Results for non‐normally distributed variables were presented as median and interquartile range (IQR). Categorical data were expressed as percentages, and comparisons of qualitative data were performed using Fisher's exact test. Diagnostic Accuracy, balanced accuracy, sensitivity, specificity, and precision were calculated based on standard definitions [24].

We chose to use Balanced Accuracy specifically because we were working with an imbalanced dataset, where one class (e.g., tumoral) was more represented than the other. Additionally, we calculated the F1 score, which represents the harmonic mean of precision and sensitivity. This metric is particularly useful for imbalanced data as it accounts for both false positives and false negatives. Agreement between Junior and Seniors' predictions was assessed using Cohen's Kappa test, according to standard definition. All tests were two‐sided, with a *p*‐value of 0.05 indicating statistical significance.

## 4 Results

### 4.1 Patients and Lesions Characteristics

Sixty‐four patients underwent the r‐EBUS‐Iriscope procedure for small PPNs during the study period. Three patients without a final diagnosis by September 2024 were excluded from analysis.

Table 1 shows the main characteristics of the PPNs in our study cohort. The average nodule size was 15 mm (long axis, IQR: 11–20 mm). The median distance from the pleura was 13 mm (IQR: 5–20 mm). On CT scans, nodules appeared solid in 48/61 (78.7%) cases, pure ground glass in 12/61 (19.6%), and mixed in one case. A bronchus sign was present in 44/61 (72%) patients.

**TABLE 1:** Nodules characteristics.

All, *n*	64
Patients with Final Diagnosis at the end of the study, *n*	61
Diagnosis obtained by r‐EBUS sampling, *n*	57
Large diameter of the nodule (mm), mean + IQR	15 (11–20)
Small diameter of the nodule (mm), mean + IQR	11 (8–14)
Distance from pleura (mm), mean + IQR	13 (5–20)
Type of nodule *n* (%)	
Solid	48 (78.7%)
Ground‐Glass	12 (19.6%)
Mixed	1 (1.6%)
Bronchus sign on r‐EBUS *n* (%)	44 (72%)

Endoscopic procedures were performed under local anaesthesia without sedation in 41/61 (67%) patients and 20/61 (33%) under general anaesthesia. r‐EBUS visualisation was successful in 90% of cases (55/61), with 30 showing centred views (49%), 25 tangential views (41%), and 6 (10%) with no r‐EBUS view. The length of the Iriscope adding procedure was about 1–3 min. No complications occurred.

A final diagnosis for lung cancer was obtained in 37/61 cases, 33/37 (89%) through endoscopic sampling, while 4/37 (11%) cases were through surgery (3 patients) or CT‐guided biopsy (1 patient). The remaining 24 non‐cancer cases were diagnosed as follows: 17 inflammatory/infectious nodules that disappeared on follow‐up imaging within 3 to 6 months, and 7 benign lesions (3 aspergillomas, 1 tuberculoma, 1 hamartoma, and 2 cases of cryptogenic organised pneumonias (COP)).

### 4.2 Iriscope Imaging Interpretation

#### 4.2.1 Human Interpretation

Seniors categorised 44/61 cases as malignant, correctly identifying 37 lung cancers (Table 2). The 7 false positives included 3 aspergillomas, 1 tuberculoma, 1 COP, and 2 inflammatory granulomas that regressed on follow‐up imaging. True Positive Rate (TPR) (Sensitivity) for the diagnosis of cancer was 100%, with a Positive Predictive Value (Precision) of 84.1%. The True Negative Rate (TNR) (Specificity) was 70.8%, and Balanced Accuracy was 85.4%. F‐1 Score was 91.4%. Seniors' interpretation was unaffected by r‐EBUS imaging type (circumferential, tangential or no image) (*p* = 0.7363).

**TABLE 2:** Human interpretation compared to final diagnosis. Expressed in percentage.

	Junior 1	Junior 2	Junior 3	Junior 4	95% CI*	Senior
Accuracy	68.9%	60.7%	65.6%	67.2%	65.6% (± 3.5%)	88.5%
Balanced accuracy	68.5%	63.9%	65.8%	68.6%	66.7% (± 2.2%)	85.4%
Specificity	66.7%	79.2%	66.7%	75.0%	71.9% (± 6.1%)	70.8%
Precision	76.5%	78.3%	75.0%	79.3%	77.3% (± 1.8%)	84.1%
Sensitivity	70.3%	48.6%	64.9%	62.2%	61.5% (± 9.0%)	100.0%
F‐1 score	73.2%	60.0%	69.6%	69.7%	68.1% (± 5.6%)	91.4%
Kappa	0.516	0.261	0.464	0.455	0.418	n/a

*Confidence Interval.

Juniors performed worse, with a True Positive Rate (TPR) (Sensitivity) of 61.5% and a Positive Predictive Value (Precision) of 77.3% (95% CI, ± 1.8%). Their True Negative Rate (TNR) (Specificity) was 71.9%, Balanced Accuracy 66.7%, and F‐1 Score 68.1% (Table 2).

In two patients, the Iriscope image was classified as malignant by both Seniors and Juniors; these 2 patients had negative endoscopic sampling for malignancy, but were finally diagnosed with lung cancer on surgery in one case and CT‐guided biopsy in the other case.

Cohen's Kappa value for the agreement between Junior physicians and Senior physicians in the interpretation of Iriscope images was 0.418, indicating moderate agreement.

#### 4.2.2 AI‐Deep Learning Model Results

The Training set included 41 patients (25 cancer cases), whereas the Test set included 20 patients (14 cancer cases).

Figure 2 shows frame‐by‐frame AI‐generated cancer probabilities from an Iriscope sequence of a malignant PPN.

Tables 3 and 4 present the performances of Deep learning according to 3 different window size analyses, corresponding to 30, 45, and 60 consecutive frames showing constant features, respectively, alongside the performances of the Senior and Junior (95% CI) groups. As can be expected, larger window sizes tend to provide a better specificity but a lower sensitivity for the diagnosis of cancer. Supporting Information Appendix A1 provides the relationship between window size and specificity.

**TABLE 3:** Human and deep learning interpretations for all 20 test patients.

	W30^a^ (%)	W45^b^ (%)	W60^c^ (%)	Senior (%)	Junior (%)
Accuracy	68.0% (± 3.9)	68.0% (± 2.4)	66.0% (± 3.7)	90%	60.0% (± 6.9)
Balanced accuracy	66.7% (± 4.0)	68.0% (± 2.1)	66.7% (± 4.5)	87.5%	63.5% (± 6.6)
Sensitivity	73.3% (± 3.3)	68.3% (± 6.1)	63.3% (± 4.0)	100.0%	45.8% (± 15.6)
Specificity	60.0% (± 3.3)	67.5% (± 6.0)	70.0% (± 9.8)	75.0%	81.2% (± 15.8)
Precision	73.3% (± 3.3)	76.1% (± 2.5)	76.6% (± 6.3)	85.7%	82.2% (± 14.0)
F1 Score	73.3% (± 3.3)	71.8% (± 3.2)	69.1% (± 2.9)	92.3%	56.6% (± 12.9)

*Note*: The 30, 45, and 60 frames window sizes correspond to fixed observation periods of 1, 1.5, and 2 s respectively. These window sizes align with the typical amount of video a physician would review to detect a tumour, allowing for a more accurate comparison between the DL models and human interpretation.

^a^ Window size of 30 frames.

^b^ Window size of 45 frames.

^c^ Window size of 60 frames.

**TABLE 4:** Human and DL interpretations for 18 test patients, with diagnosis obtained by r‐EBUS sampling.

	W30^a^ (%)	W45^b^ (%)	W60^c^ (%)	Senior (%)	Junior (%)
Accuracy	73.3% (± 4.1)	71.1% (± 8.0)	67.8% (± 6.4)	88.9%	65.3% (± 9.3)
Balanced accuracy	72.0% (± 3.8)	71.5% (± 7.2)	68.5% (± 5.7)	87.5%	66.9% (± 8.6)
Sensitivity	84.0% (± 7.8)	68.0% (± 1.4)	62.0% (± 11.4)	100.0%	52.5% (± 20.2)
Specificity	60.0% (± 4.9)	75.0% (± 0.0)	75.0% (± 0.0)	75.0%	81.3% (± 15.8)
Precision	72.4% (± 2.4)	76.7% (± 3.7)	75.1% (± 3.3)	83.3%	81.0% (± 15.3)
F1 Score	77.6% (± 4.3)	71.6% (± 9.6)	67.6% (± 8.2)	90.9%	61.0% (± 15.0)

*Note*: The 30, 45, and 60 frames window sizes correspond to fixed observation periods of 1, 1.5, and 2 s respectively. These window sizes align with the typical amount of video a physician would review to detect a tumour, allowing for a more accurate comparison between the DL models and human interpretation.

^a^ Window size of 30 frames.

^b^ Window size of 45 frames.

^c^ Window size of 60 frames.

Using a 45 frames window size, True Positive Rate (TPR) (Sensitivity) of Deep Learning's Iriscope classification was 68% (95% CI, ± 7.8%) for the diagnosis of cancer with a Positive Predictive Value (Precision) of 76.7% (95% CI, ± 2.4%). The True Negative Rate (TNR) (Specificity) was 75% (95% CI, ± 0.0%). Balanced Accuracy was 71.5% (95% CI, ± 7.2%). F1 Score was 71.6% (95% CI, ± 9.6%).

Compared to human performances, the AI model outperformed Juniors, but did not surpass the Seniors.

Both humans and DL performed better in cancer prediction when the Gold Standard was based on the final diagnosis (20 patients). This diagnosis was determined by considering not only the endoscopic sampling results but also additional sampling procedures (e.g., surgery or CT‐guided biopsy) in cases where endoscopic sampling was inconclusive (Tables 3 and 4).

## 5 Discussion

The present study shows that direct endoscopic visualisation of PPNs smaller than 2 cm is feasible using a miniaturised video endoscopy probe, and that endoscopic imaging of these peripheral lung lesions can predict malignancy with high accuracy, both by human eyes and a deep learning‐generated prediction model.

To our knowledge, before our recent short publication using Iriscope technology [11], in vivo endoscopic imaging of small PPNs had only been reported at the microscopic level using confocal laser fluorescence endomicroscopy and in situ methylene blue imaging, which, at present, cannot be used in everyday practice [25, 26].

Recently, Kinoshita used a 0.97 mm fiberoptic probe, ex vivo in three cases, and visualised the peripheral tumour in one [27], but the technology has not yet been assessed in vivo. Other technologies, such as ultrathin bronchoscopes or robotic‐assisted bronchoscopy, that may allow direct vision and sampling of lung nodules are currently limited to the middle third of the lung [28, 29] and therefore cannot yet be considered as true “peripheral bronchoscopy” imaging techniques. Although the scopes may not advance to the outer third (although in many cases this is possible), the tools such as rEBUS, needles and cryoprobes can essentially reach the pleura and allow sampling of very peripheral lesions. However, these methods do not allow a direct vision of the nodule in the same way the Iriscope can.

Our study is therefore the first to assess the use of a miniaturised probe for direct visualisation and prediction of cancer in a large series of patients with subpleural PPNs smaller than 2 cm.

Using our previous descriptors of Iriscope imaging for peripheral cancer lesions, experienced bronchoscopists were able to differentiate malignant from non‐malignant nodules with a positive predictive value and a balanced accuracy both reaching 85%. This confirms the results of our published study in terms of the performances of the technique, as well as the validity of the endoscopic descriptors of cancer we previously defined for the peripheral bronchi [11].

In this study, due to the small number of GGos, we were not able to make a difference between GGos and solid nodules with Iriscope. This question requires future studies. Interestingly, the ability of experienced endoscopists to recognise cancer from Iriscope images was not affected by the position of the r‐EBUS catheter relative to the centre of the lesion, as it did not differ when comparing centred or tangential r‐EBUS images. The technique was also successful in six cases where no characteristic image could be seen on r‐EBUS. While this cannot be proven definitively by this series, it may indicate that the visualisation of the lesion using a miniaturised videoprobe improves the precision of the sampling in cases where r‐EBUS provides tangential imaging or fails to localise the lesion, situations that are known to be associated with a lower rate of diagnostic biopsies [30, 31].

In line with these findings, r‐EBUS + Iriscope allowed the visualisation and sampling of six lesions appearing as ground glass opacities and of 12 lesions without bronchus sign on CT, for which bronchoscopy sampling is usually challenging [32, 33].

On the other hand, junior bronchoscopists had more difficulties to recognise the endoscopic signature of cancer using Iriscope imaging. This could be explained by the fact that they are not yet fully trained to recognise subtle bronchial mucosal changes, especially within the non‐cartilaginous small bronchioles or due to the lower definition of the Iriscope image (400 × 400 pixels) compared to the standard video bronchoscopes. If the DL component did not outperform the experienced bronchoscopist’ performance, the difference between junior and experienced bronchoscopists emphasises the need for an AI‐DL help to differentiate cancer and benign lesions when using Iriscope for trainees. The improvement of the AI‐DL model may help spreading navigation bronchoscopy techniques, and especially Iriscope, even in low volume centres.

While the integration of AI has already shown some potential in identifying lung diseases using r‐EBUS [34] images or Rapid On Site Cytology images [35], only one published study tested AI on bronchoscopy images [36]. In this recent study, Vu VG et Al. trained AI to recognise lung cancer endoscopic images, recorded from 4 to 6 mm bronchoscopes in large proximal bronchi. The endoscopic images were extracted from 208 bronchoscopy videos of 106 lung cancer patients and 102 individuals who did not have lung cancer. Ten high‐quality images per case were selected by senior bronchoscopists with manual delineation of the visible tumours. The study was conducted with a training set of 237 images and a test set of 263 images, using a convolutional neural network‐based model. The mean accuracy for cancer classification was 0.85.

With a different method for image processing, applied to small PPNs, the DL method used in our series provided results similar to Vu's report in large bronchi, taking into account that, by definition, expert endoscopist in Vu's study classified tumoral images with 100% accuracy. By contrast, our study was performed on an unselected set of 62,000 images or frames, which represents the entirety of the Iriscope video recording for each patient. This frame‐by‐frame approach, which could be integrated in the future into the Iriscope imaging technique in vivo, has the potential to provide additional information to the bronchoscopist in real time for more accurate cancer prediction and precise bronchial sampling.

While the DL model presented here is not yet at the level of the senior endoscopists' interpretation, it outperformed the junior physicians across several relevant metrics.

Interestingly, both human bronchoscopists, regardless of experience level, and the Deep learning machine model performed better in the prediction of cancer when the final diagnosis was considered, that is, including cancer patients with a negative endoscopic biopsy. This can be explained by the difficulties in obtaining adequate tissue sampling from a small peripheral nodule during the peripheral bronchoscopy [37]. This suggests that in vivo endoscopic detection of a tumoral aspect with negative sampling could improve decision‐making for patients with PPNs, particularly when deciding whether a rapid invasive diagnostic approach is warranted. Conversely, a benign appearance may help to avoid unnecessary resections of benign lesions [38, 39]. Future studies might evaluate if a composite endpoint, associating the Iriscope and the CT patterns, would be a better predictor than CT alone for progression of high‐risk lesions.

Our study has some limitations. The sample size was relatively small, and the study was conducted at a single centre, limiting the generalisability of the findings. Presumably, even though a bronchus sign was not present in all cases, this technique still requires an airway, even if it is tiny and not seen on CT, leading to the lesion; otherwise, the Iriscope will have no way to get to the lesion, as the Iriscope may not be able to traverse the pulmonary parenchyma.

Additionally, the reliance on subjective interpretation of Iriscope images, despite the use of standardised categories, likely introduced variability. Furthermore, the training set for the AI model was also limited in size, which may have constrained its performance. Future studies could include training the AI with larger datasets, as well as incorporating more advanced techniques such as recurrent neural networks to account for the temporal dimension of video sequences.

In conclusion, Iriscope could be a valuable tool in PPNs management, especially for experienced operators. Applied to Iriscope images, AI could enhance overall performance of less experienced physicians in diagnosing malignancy.

## Author Contributions

**Edoardo Amante:** conceptualization (equal), data curation (lead), formal analysis (equal), funding acquisition (equal), investigation (equal), methodology (equal), project administration (equal), resources (equal), software (equal), supervision (equal), validation (equal), visualization (equal), writing – original draft (equal), writing – review and editing (equal). **Robin Ghyselinck:** conceptualization (equal), data curation (equal), formal analysis (lead), funding acquisition (equal), investigation (equal), methodology (equal), project administration (equal), resources (equal), software (lead), supervision (equal), validation (equal), visualization (equal), writing – original draft (equal), writing – review and editing (equal). **Luc Thiberville:** conceptualization (equal), data curation (equal), formal analysis (equal), funding acquisition (equal), investigation (equal), methodology (equal), project administration (equal), resources (equal), software (equal), supervision (equal), validation (equal), visualization (equal), writing – original draft (equal), writing – review and editing (lead). **Rocco Trisolini:** conceptualization (equal), formal analysis (equal), investigation (equal), supervision (equal), validation (equal), visualization (equal), writing – original draft (equal), writing – review and editing (equal). **Florian Guisier:** investigation (equal), project administration (equal), validation (equal), visualization (equal), writing – original draft (equal), writing – review and editing (equal). **Valentin Delchevalerie:** formal analysis (equal), methodology (equal), software (equal), validation (equal), visualization (equal), writing – original draft (equal), writing – review and editing (equal). **Bruno Dumas:** formal analysis (equal), investigation (equal), methodology (equal), supervision (equal), validation (equal), visualization (equal), writing – original draft (equal), writing – review and editing (equal). **Benoît Frénay:** formal analysis (equal), investigation (equal), methodology (equal), software (equal), supervision (equal), validation (equal), visualization (equal), writing – original draft (equal), writing – review and editing (equal). **Inès Duparc:** data curation (equal), formal analysis (equal), investigation (equal), resources (equal), validation (equal), visualization (equal), writing – review and editing (equal). **Nicolas Mazellier:** data curation (equal), visualization (equal), writing – review and editing (equal). **Cecile Farhi:** data curation (equal), validation (equal), visualization (equal), writing – review and editing (equal). **Christophe Jubert:** data curation (equal), validation (equal), writing – review and editing (equal). **Mathieu Salaün:** conceptualization (equal), data curation (equal), formal analysis (equal), formal analysis (equal), funding acquisition (equal), funding acquisition (equal), investigation (equal), investigation (equal), methodology (equal), methodology (equal), project administration (equal), project administration (equal), resources (equal), resources (equal), software (equal), software (equal), supervision (equal), supervision (equal), validation (equal), validation (equal), visualization (equal), visualization (equal), writing – original draft (equal), writing – original draft (equal), writing – review and editing (equal), writing – review and editing (equal). **Samy Lachkar:** conceptualization (lead), data curation (lead), formal analysis (equal), funding acquisition (equal), investigation (equal), methodology (equal), project administration (equal), resources (equal), software (equal), supervision (equal), validation (equal), visualization (equal), writing – original draft (equal), writing – review and editing (equal).

## Ethics Statement

The study protocol was approved by the Institutional review Board of Rouen University Hospital (E2024‐72). Research followed the European Directive 2014/536/EU and the French law 2012‐300 on biomedical research. Consent was not required for retrospective data analysis under French law.

## Conflicts of Interest

S.L. received consulting fees in Lys medical, Olympus and Fujifilm. The remaining authors declare no conflicts of interest.

## Supporting information


**Appendix A1.** Supporting Information.

Visual Abstract

## Data Availability

The data that support the findings of this study are not publicly available due to their containing information that could compromise the privacy of research participants but are available from Samy Lachkar on request.
