# Supplementary material for: Human and Deep Learning Predictions of Peripheral Lung Cancer Using a 1.3 mm Video Endoscopic Probe
Source: Respirology. 2025 May 28;30(9):861–70. doi: 10.1111/resp.70057 (PMC12437996; doi:10.1111/resp.70057)
Supplement: Supplementary file 1 — Appendix A1. Supporting Information. [file RESP-30-861-s001.docx]

# 1. Classification Performance of Humans

This section deals with the performance of humans on images classification task.

First, the tools used in the analysis are mentioned and the selected performance metrics are shortly described. Second, the results of each individual Junior physician, together with their 95% confidence interval are presented, and compared against the performance of the Seniors.

## 1.1 Statistical analysis tools and Performance Metrics

### 1.1.1 Statistical analysis tools

The below formulas have been computed using the Python 3.10 programming language and the following libraries:

- scipy 1.14.1
- scikit-learn 1.5.1
- Pandas 2.2.2
- Numpy 2.1.0

Those libraries are standard practice in machine learning and data science applications.

### 1.1.2 Performance Metrics

The performance metrics are presented below. Note that when considering interns and experts, the final diagnosis comes from all the 61 patients analyzed, such that there is only one possible answer per patient. This is not necessarily the case for the deep learning model, where a prediction per frame is also made.

$$Accuracy = \frac{\left( True Positives+True Negatives \right)}{\left( Total Number of Samples \right)}$$

Accuracy is a measure of the proportion of correct predictions (both true positives and true negatives) out of the total predictions. This metric indicates the overall performance but may not fully capture performance when class imbalances exist. For example, a situation of binary classification where one class represents 60% of the data and the other represents 40% of the data would already be considered imbalanced.

$$Balanced Accuracy =\frac{\left( Sensitivity +Specificity \right)}{2}$$

Balanced accuracy is the average of sensitivity and specificity for each class. This ensures that performance is weighted equally across classes and is a better representation of performance when dealing with imbalanced data.

$$Sensitivity =\frac{True Positives}{\left( True Positives+ False Negatives \right)}$$

Sensitivity (True Positive Rate or Recall) is a measure of the ratio of true positives correctly identified by the model.

$$F1 =2\cdot\frac{\left( precision \cdot sensitivity \right)}{\left( precision + sensitivity \right)}$$

The F1 score is the harmonic mean of precision and sensitivity. This metric is useful in the presence of imbalanced data, as it considers false positives and false negatives.

$$\Pr ecision = \frac{True Positives}{\left( True Positives + False Positives \right)}$$

Precision (Positive Predicted Value) is a measure of the proportion of positive predictions that are actual true positives.

$$Specificity=\frac{True Negatives}{True Negatives + False Positives}$$

Specificity (True Negative Rate) is a measure of the proportion of actual negatives that are correctly identified.

$$Kappa=\frac{1}{n}\cdot\sum_{1}^{n} \frac{P\left( O_{i}=E \right)-Pe}{1 - P_{e}}$$

Cohen’s Kappa test is a measure of the agreement between the predictions made by a reference observer and each individual observer ($O_{i}$), where $P_{e}$ is the expected agreement between E and $O_{i}$ by chance.

## 1.2 Results

**e-Table 1** shows the classification results of each individual Junior physician (identified by a unique identifier from 1 to 4 in the table) together with their 95% confidence interval (CI), and the performance of the Senior, across the selected performance metrics.

### **e-Table 1: Metrics for Each Column Compared to Final Diagnosis**

| **Metric** | **1** | **2** | **3** | **4** | **95% CI*** | **Senior** |
| --- | --- | --- | --- | --- | --- | --- |
| Accuracy | 68.9% | 60.7% | 65.6% | 67.2% | 65.6% (+/- 3.5%) | 88.5% |
| Balanced Accuracy | 68.5% | 63.9% | 65.8% | 68.6% | 66.7% (+/- 2.2%) | 85.4% |
| Specificity | 66.7% | 79.2% | 66.7% | 75.0% | 71.9% (+/- 6.1%) | 70.8% |
| Precision | 76.5% | 78.3% | 75.0% | 79.3% | 77.3% (+/- 1.8%) | 84.1% |
| Sensitivity | 70.3% | 48.6% | 64.9% | 62.2% | 61.5% (+/- 9.0%) | 100.0% |
| F-1 | 73.2% | 60.0% | 69.6% | 69.7% | 68.1% (+/- 5.6%) | 91.4% |
| Kappa | 51.6% | 26.1% | 46.4% | 45.5% | 41.8% | n/a |

### **Except for Kappa where only the average is presented.*

# 2. Classification Performance of Deep Learning

## 2.1 Tools

The original videos have been processed using the C++ 23 programming language, and the following packages:

- gstreamer 1.0
- OpenCV 4.10.0

For training the deep learning models, the Python 3.10 programming language has been used with the following libraries:

- PyTorch 2.4.0
- Torchvision 0.19.0
- scikit-learn 1.5.1
- Pandas 2.2.2
- Numpy 2.1.0

## 2.2 Data source

The original 61 videos are recorded with a resolution of 400 x 400 pixels at a frame rate of $30$frames per second. Each video has been transformed into individual frames, where one second of video yields 30 frames of 400 x 400 pixels. This process results in the creation of a dataset comprised of 62072 frames, separated into 61 videos, one per patient. Each individual frame was assigned with a binary annotation: “non- tumoral” or “tumoral”. The latter is either showing *whitish friable tumoral tissue*, *fish flesh* or *mucosal outgrowth^a^*. The classes exhibit some level of imbalance since 60.1% of the patients and 38.0% of individual frames show a “tumoral” aspect, against 39.9% of the patients and 62.0% of individual frames that are “non-tumoral”. In addition, the lengths of the individual videos varied significantly, with an average of 34 seconds (1017 frames) and a standard-deviation of 26 seconds (795 frames).

## 2.3 Methodology

In this work, a ResNet-50^[[1]](#footnote-1)^ model pre-trained on the ImageNet^[[2]](#footnote-2)^ dataset to perform binary classification of individual frames extracted from videos. This is standard practice in deep learning pipelines that deal with such computer vision tasks. The objective of the deep learning model is to learn representations of the images in the dataset and classify each individual image as showing a tumor or not by optimizing a binary cross-entropy loss that captures the performance of the model.

$$Loss = \frac{-1}{N}\cdot\sum_{1}^{N} \left[ y_{i} \cdot\log\left( p_{i} \right) + \left( 1-y_{i} \right) \cdot\log\left( 1-p_{i} \right) \right]$$

Where $y_{i}$is the ground truth (1 if tumor, otherwise 0) and $p_{i}$ is the probability assigned by the model to the $i_{th}$ example.

### **2.3.1 Data Splitting**

The data was split using randomized sampling of patients to ensure a meaningful division into training and testing sets without having a single image from the same patient in both training and testing sets. In this way, data leakage is not possible, and this bias is never exhibited in the results.

The split, presented in **e-Table 2**, is designed to provide a comprehensive representation of the data, both in terms of number of patients, number of frames, and class distribution. Only patients for which final diagnosis was obtained through endoscopic sampling with r-EBUS are included in this test set. As such, the patient splitting between the two sets in Table 2 serves as a basis for the subsequent split in Table 3. **In e-Table 3**, four additional patients have been included, for which the final diagnosis was obtained on other methods than endoscopy sampling with r-EBUS. Those patients have been randomly assigned to either training or testing set.

**e-Table 2**: Train and Test dataset split description – 57 patients with final diagnosis obtained through endoscopic sampling with r-EBUS are included

| **Metric** | **Train Set** | **Test Set** |
| --- | --- | --- |
| Frame % | 70.9% | 29.1% |
| Patient % | 67.9% | 32.1% |
| Tumoral class % of frames (% of patients in set) | 28.9% (57.9%) | 41.1% (55.6%) |
| Non-tumoral class % (% of patients in set) | 71.1% (42.1%) | 58.9% (44.4%) |

**e-Table 3**: Train and Test dataset split description – All 61 patients are included

| **Metric** | **Train Set** | **Test Set** |
| --- | --- | --- |
| Frame % | 71.5% | 28.5% |
| Patient % | 67.2% | 32.8% |
| Tumoral class % of frames (% of patients) | 28.9% (61.0%) | 39.4% (60.0%) |
| Non-tumoral class % | 71.1% (39.0%) | 60.6% (40.0%) |

### **2.2.2 Model Training**

The following training hyper-parameters for the ResNet-50 model were selected:

- **Number of epochs**: 200. This means that the model will go through all the data 200 times during the full training process.
- **Batch size**: 256. The model will process 256 images before updating its weights.
- **Learning rate**: 1e-4. This parameter is the magnitude by which the weights of the model are adjusted relative to the loss gradient during training.
- **Optimizer**: AdamW^[[3]](#footnote-3)^ with weight decay of 1e-4. This applies weight decay to the weights to improve regularization and prevent overfitting.
- **Scheduler**: Cosine annealing^[[4]](#footnote-4)^ . The learning rate is gradually decreasing during training, following a cosine curve. This ensures smoother convergence.

The following transformations are applied to the data during training:

- A resizing of the images from 400 x 400 pixels to 256 x 256 pixels.
- Then, a random crop from 256 x 256 pixels to 224 x 224 pixels.
- A random rotation of a multiple of 90° is applied, with an equal probability of selecting any multiple between 0 and 270°.
- Subsequently, a random flip and mirror is used, with 50% probability of having a horizontal or vertical flip.
- Shearing is applied.
- A gaussian blur.
- Finally, the image is transformed into a tensor.

The application of these randomized transformations is similar to artificially increasing the size of the training dataset and is a standard practice.

For the test data, we resize the images from 400 x 400 pixels to 224 x 224 pixels and transform them into a tensor.

Using grid search, we experimented with the above-mentioned hyper-parameters, but the performance was lower.

### **2.2.3 Handling Class Imbalance**

Given the imbalanced nature of the dataset, we employ an imbalanced sampler during training. This sampler ensures that training batches are composed of approximately 50% tumoral and 50% normal frames, allowing the model to learn more effectively from both classes and reducing its bias toward the majority class.

This methodology is designed to mitigate class imbalance, maintain generalization to unseen data, and ensure a balanced evaluation across patients and frames.

## 2.2.3 Results

After the training process is complete, the model showing the lowest test loss is selected. Five models are independently trained and tested, where only they initialization differed. In this way, it is possible to present 95% confidence intervals on the results. The metrics presented in **e-Table 4** are obtained using the predictions against the ground truth labels on all the frames from full test set. The metrics from **e-Table 5** only include patients where the final diagnosis was obtained through endoscopic biopsy with r-EBUS. Although these results cannot be directly compared against those of humans (see Section 1), they are meaningful in that they establish a baseline for future work using deep learning techniques. Indeed, humans only provide one answer per patient whereas deep learning model provides one answer per video frame.

e-Table 4 - All 20 test patients are included

| **Metric** | **Value (+/- 95% IC)** |
| --- | --- |
| Accuracy | 70.6% (+/- 0.8%) |
| Balanced Accuracy | 69.2% (+/- 1.8%) |
| Precision | 62.3% (+/- 0.6%) |
| Sensitivity | 62.7% (+/- 6.6%) |
| Specificity | 75.8% (+/- 3.1%) |
| F1-Score | 62.5% (+/- 3.1%) |

**e-Table 5** – 18 test patients with final diagnosis obtained through endoscopic biopsy with r-EBUS are considered

| **Metric** | **Value (+/- 95% IC)** |
| --- | --- |
| Accuracy | 62.7% (+/- 1.3%) |
| Balanced Accuracy | 61.1% (+/- 1.7%) |
| Precision | 54.8% (+/- 1.4%) |
| Sensitivity | 52.0% (+/- 5.0%) |
| Specificity | 70.2% (+/- 2.2%) |
| F1-Score | 53.3% (+/- 3.2%) |

**Discussion** : The performance shows a balanced accuracy of 69.2% for the model using data from all patients against 61.1% for the model using only patients that had a final diagnosis through endoscopic biopsy with r-EBUS (*it has 4 fewer patients overall, 2 in its training set and 2 in its test set*). This implicates that a simple deep learning model is already capable of classifying individual frames, in particular, by weighting equally errors made on positive than on the negative class. This analysis is further refined in the next section, where a per-patient analysis is performed.

# 3. Comparison of Humans and Machine Learning Performance

The results from humans presented in Section 1 are obtained by comparing their predictions per patient, on the full set of 61 patients or on the set that includes only 57 patients in which a final diagnosis was obtained through endoscopy sampling.

The results from deep learning presented in Section 2 are obtained by comparing the model’s predictions per frame, on the test set of 18 to 20 patients.

To close the gap, it is important to compare the model’s predictions per patient (not per frame), against the predictions of humans on these same patients (i.e. on the same 18 or 20 patients from the test set).

## 3.1 Methodology for Machine Learning

### 3.1.1 Outliers smoothing using ARIMA

Since the focus is on evaluating the predictions for entire patients rather than individual frames, a second stage of analysis is needed. The probabilities of the deep learning model are smoothed using ARIMA. This smoothing allows us to mitigate the frame-by-frame noise and produce more reliable predictions that represent the overall trend across a sequence of frames.

**e-Image 1** shows an illustration of model’s predicted probability (y-axis) against frame number (x-axis). Areas in green indicate a correct prediction by the model, and areas in red indicate a wrong prediction by the model. The actual labels are shown below the x-axis. The second graph provides the same information, with a smoothing on the probability using ARIMA.

**e-Image 1** Plot of model’s predictions against frame number - before and after smoothing


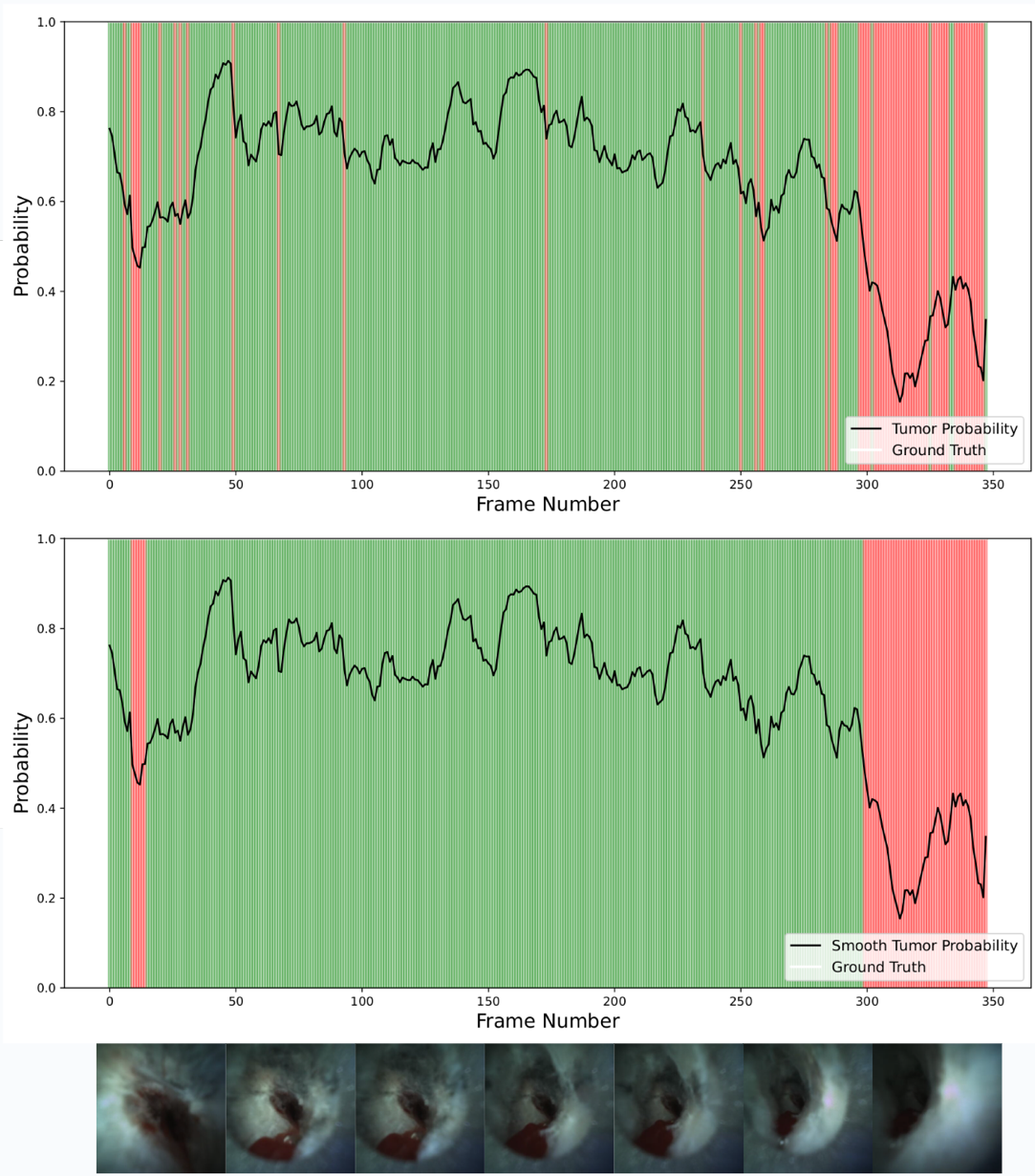


### 3.1.2 Final prediction per patient

Next, a sliding window across frames was defined. If for all frames in the sliding window, only “tumoral” were predicted, then the prediction of the deep learning model is “tumoral”. If there is no consecutive window within which “tumoral” are predicted, then the deep learning model’s prediction is “non-tumoral”.

Because this approach is sensitive to the window size, an open interval between 1 and 300 frames was chosen. Within that open interval, each value was used as the window size, and all performance metrics were computed. Note that 1 frame corresponds to about 1/30th of a second, and 300 frames corresponds to 10 seconds.

For the sake of our analysis, a window size between 30 and 60 frames was selected, which represents what a human observer would typically review—about 1 to 2 seconds worth of footage to detect the presence of a tumor.

## 3.2 Performance

**e-Table 3** shows the performance of the ResNet-50 models with 95% IC, the performance of the Senior physicians, and the performance of Junior physicians (95% IC) across all metrics for difference window size. Each model is labeled by its window size: W20, W40, W60.

**e-Table 6** – Includes all 20 test patients

|  | W30 (in %) | W45 (in %) | W60 (in %) | Expert (in %) | Intern (in %) |
| --- | --- | --- | --- | --- | --- |
| Accuracy | 68.0 (+/- 3.9) | 68.0 (+/-2.4) | 66.0(+/-3.7) | 90 | 60.0 (+/- 6.9) |
| Balanced Accuracy | 66.7 (+/-4.0) | 68.0 (+/- 2.1) | 66.7(+/- 4.5) | 87.5 | 63.5(+/-6.6) |
| Sensitivity | 73.3 (+/- 3.3) | 68.3(+/-6.1) | 63.3(+/-4.0) | 100.0 | 45.8 (+/- 15.6) |
| Specificity | 60.0 (+/- 3.3) | 67.5 (+/- 6.0) | 70.0(+/- 9.8) | 75.0 | 81.2 (+/- 15.8) |
| Precision | 73.3 (+/- 3.3) | 76.1(+/- 2.5) | 76.6(+/- 6.3) | 85.7 | 82.2(+/- 14.0) |
| F1 Score | 73.3 (+/- 3.3) | 71.8(+/- 3.2) | 69.1(+/- 2.9) | 92.3 | 56.6(+/-12.9) |

**e-Table 7** – Includes only 18 patients with final diagnosis obtained through endoscopic sampling with r-EBUS

|  | W30 (in %) | W45 (in %) | W60 (in %) | Expert | Intern (in %) |
| --- | --- | --- | --- | --- | --- |
| Accuracy | 73.3 (+/- 4.1) | 71.1(+/-8.0) | 67.8 (+/- 6.4) | 88.9 | 65.3 (+/- 9.3) |
| Balanced Accuracy | 72.0 (+/- 3.8) | 71.5(+/- 7.2) | 68.5 (+/-5.7) | 87.5 | 66.9 (+/- 8.6) |
| Sensitivity | 84.0 (+/- 7.8) | 68.0(+/-1.4) | 62.0(+/- 11.4) | 100.0 | 52.5 (+/- 20.2) |
| Specificity | 60.0 (+/- 4.9) | 75.0 (+/-0.0) | 75.0(+/- 0.0) | 75.0 | 81.3 (+/- 15.8) |
| Precision | 72.4 (+/- 2.4) | 76.7 (+/- 3.7) | 75.1(+/-3.3 ) | 83.3 | 81.0 (+/- 15.3) |
| F1 Score | 77.6(+/- 4.3) | 71.6 (+/- 9.6) | 67.6(+/- 8.2) | 90.9 | 61.0 (+/- 15.0) |

The window sizes of 30, 45, and 60 that are presented in **Table 6 and 7** represent a fixed observation period of 1, 1.5, and 2 seconds respectively. If window size is changed to different values, **Image 2** shows that the result for any integer on the open interval from 30 to 60 would perform similarly.

**e-Image 2** – Balanced accuracy per window size


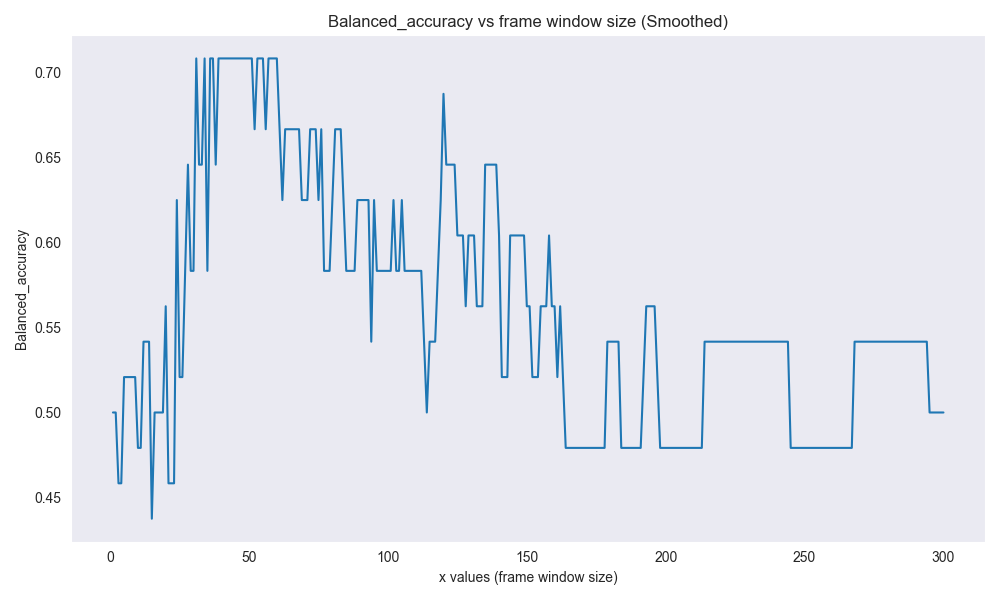


**e-Image 3** : Specificity for cancer diagnosis in relation with frame window size


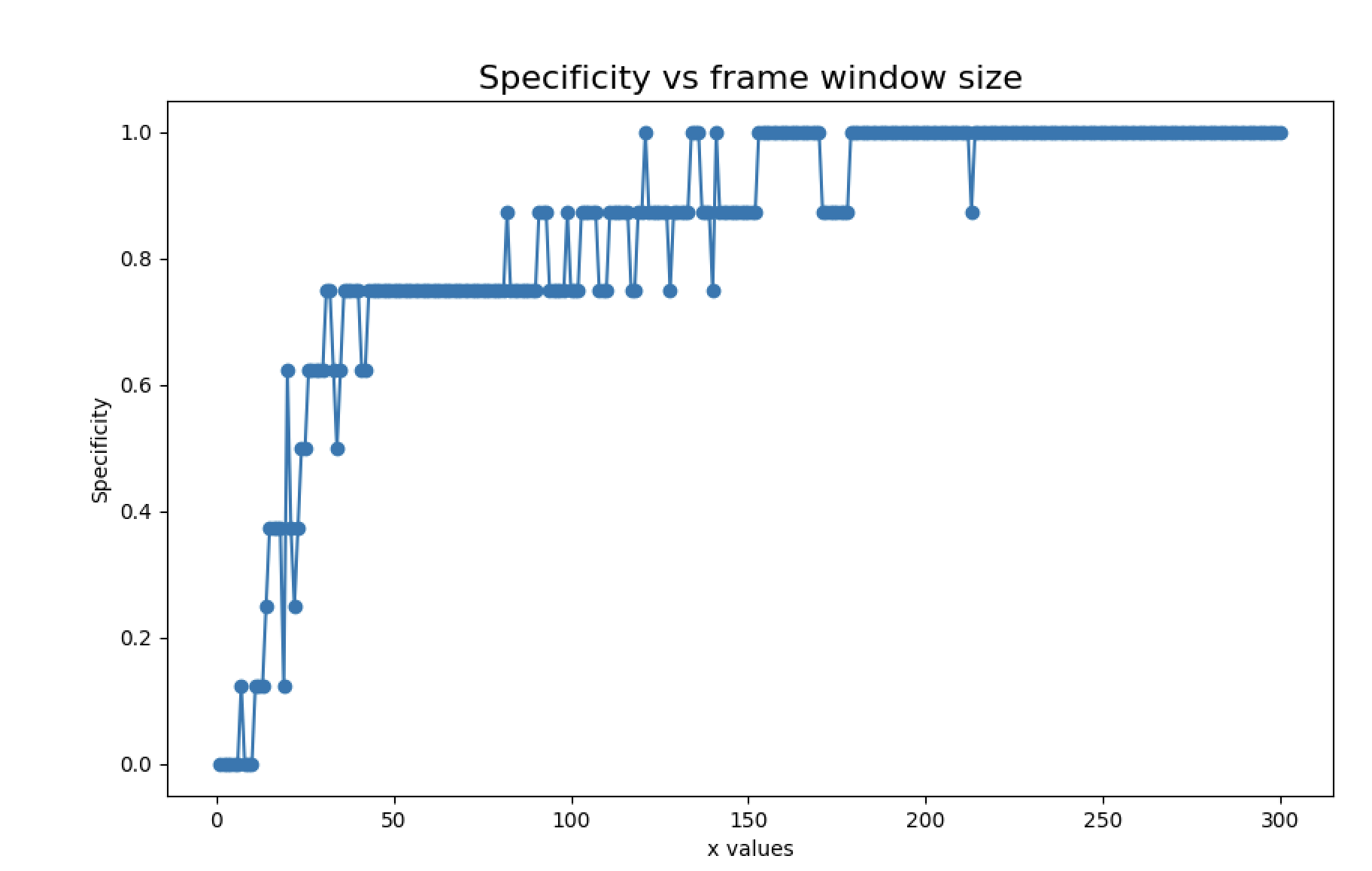


e-Image 4 : Sensitivity for cancer diagnosis in relation with frame window size


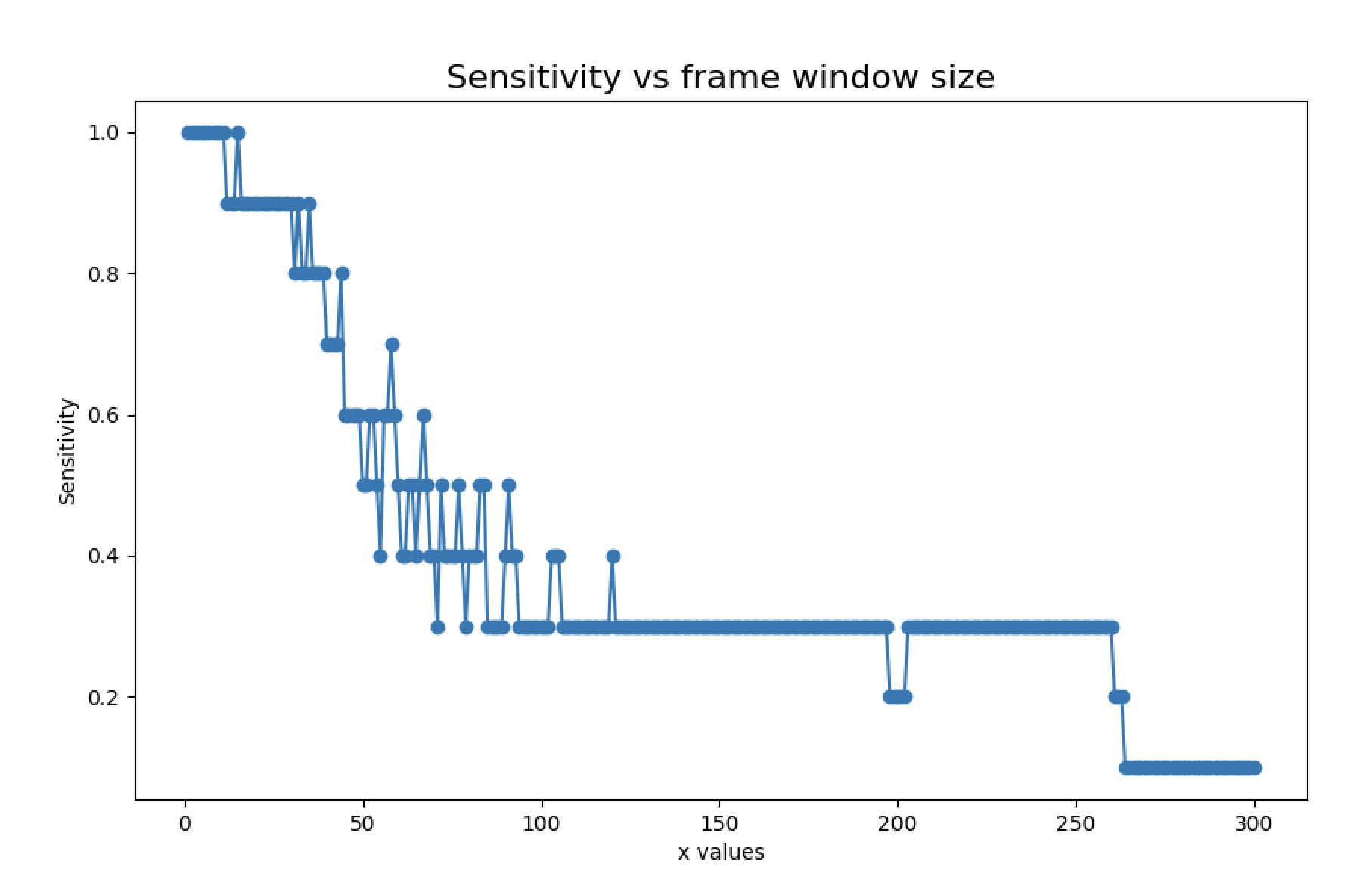


1. https://www.cv-foundation.org/openaccess/content_cvpr_2016/papers/He_Deep_Residual_Learning_CVPR_2016_paper.pdf [↑](#footnote-ref-1)
2. <https://ieeexplore.ieee.org/document/5206848>

   ^a^ Lachkar S,  Duparc I,  Piton N,  Dantoing E,  Thiberville L,  Guisier F, et al.  Direct endoscopic visualization of small peripheral lung nodules using a miniaturized videoendoscopy probe. *Respirology*.  2024; 29(10): 914–917. <https://doi.org/10.1111/resp.14798> [↑](#footnote-ref-2)
3. <https://www.semanticscholar.org/paper/Decoupled-Weight-Decay-Regularization-Loshchilov-Hutter/d07284a6811f1b2745d91bdb06b040b57f226882> [↑](#footnote-ref-3)
4. <https://arxiv.org/abs/1711.05101> [↑](#footnote-ref-4)
