# Supplementary material for: Human and Deep Learning Predictions of Peripheral Lung Cancer Using a 1.3 mm Video Endoscopic Probe
Source: Respirology. 2025 May 28;30(9):861–70. doi: 10.1111/resp.70057 (PMC12437996; doi:10.1111/resp.70057)
Supplement: Supplementary file 2 — Visual Abstract [file RESP-30-861-s002.pdf]

Human and deep learning predictions of peripheral lung cancer using a 1.3 mm video endoscopic probe

- Peripheral pulmonary nodules (<20 mm) are challenging to diagnose.
- IRISCOPE® enables direct endoscopic visualization.

Aim: Evaluate human (junior vs senior) and deep learning performance in classifying peripheral lesions (malignant vs non-malignant) using IRISCOPE® images.

VISUAL COMPARISON

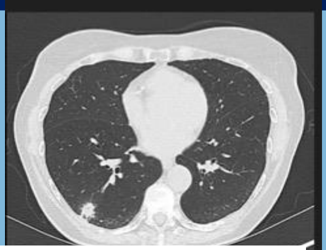

CT Scan

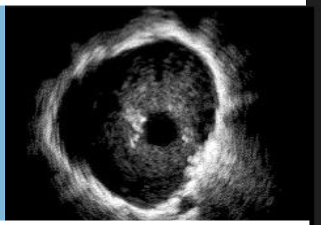

rEBUS signal

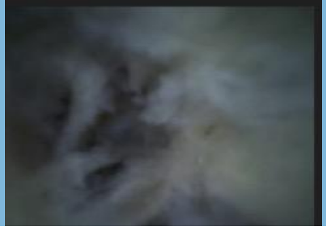

IRISCOPE ® image of a tumor

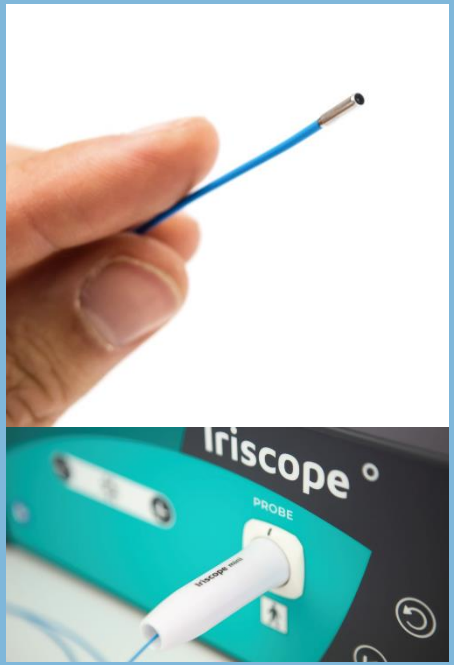

IRISCOPE® provided lesion visualization in all cases, including those with negative or tangential r-EBUS signal.

Deep Learning outperformed juniors (accuracy 71.5% vs 66.9%) but not seniors (accuracy 87.5%)

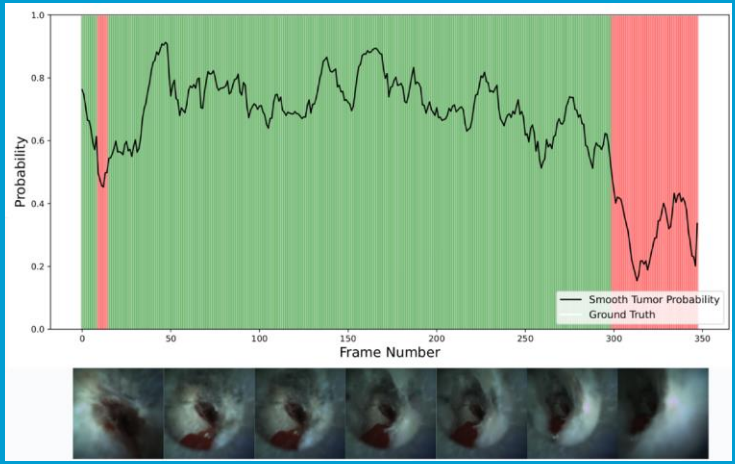

IRISCOPE® and deep learning together may improve the management of peripheral lesions.

Respirology

Human and Deep Learning Predictions of Peripheral Lung Cancer Using a 1.3 mm Video Endoscopic Probe. Amante et al., Respirology, 2025; DOI 10.1111/resp.70057
